# Financial Toxicity Associated with Biological Medicines: A Scoping Review

**DOI:** 10.1002/cpt.70278

**Published:** 2026-04-03

**Authors:** Laura Sara Maria Saarukka, Kamilla Yamileva, Kari Linden, Anniina Simonen, Anna‐Riia Holmström

**Affiliations:** ^1^ Faculty of Social Sciences University of Helsinki Helsinki Finland; ^2^ Faculty of Pharmacy University of Helsinki Helsinki Finland; ^3^ Research Unit University Pharmacy Helsinki Finland; ^4^ Faculty of Veterinary Medicine University of Helsinki Helsinki Finland

## Abstract

Financial toxicity, defined as the economic burden experienced by patients due to medication and other healthcare costs and their consequences, such as material, psychosocial and behavioral effects, represents a significant concern for individuals receiving biological medicines. This scoping review synthesizes current evidence on financial toxicity connected to biological therapies and proposes policy interventions to mitigate its impact, while identifying key research gaps. A comprehensive literature search was conducted across databases Scopus, Medline, Web of Science, CINAHL, and EMB Reviews. The initial set of articles was screened in accordance with the PRISMA framework using Covidence software. After title/abstract and full‐text screening, 24 studies were included in the final analysis. Data were synthesized guided by Witte's framework of financial toxicity, which distinguishes between objective financial burden (direct and indirect treatment costs) and subjective financial distress (material, psychosocial and behavioral effects). Findings indicate that high out‐of‐pocket expenses are the primary driver of financial toxicity among patients using biological medicines. These costs are often exacerbated by indirect expenses such as lost income, as well as insurance‐related barriers including high co‐payments. Although several studies acknowledged the subjective financial distress related to financial toxicity, this dimension was underreported, with few studies incorporating patient‐reported outcomes or validated quality‐of‐life measures. This review reinforces the multifaceted nature of financial toxicity in the context of biological therapies and highlights the urgent need for support systems that address both economic and psychosocial challenges faced by patients. Future research should prioritize strategies to improve access to treatment while minimizing the financial burden.

**Study Highlights**

**WHAT IS THE CURRENT KNOWLEDGE ON THE TOPIC?**

Financial toxicity is acknowledged as a concern for individuals with chronic illnesses requiring long‐term treatment. Patients using biological medicines face substantial out‐of‐pocket costs despite policy efforts to reduce costs.

**WHAT QUESTION DID THIS STUDY ADDRESS?**

What is the current evidence on financial toxicity connected to biological therapies?

**WHAT DOES THIS STUDY ADD TO OUR KNOWLEDGE?**

This scoping review synthesis reveals the complex and multifaceted nature of financial toxicity linked to the use of biological medicines, encompassing direct and indirect treatment costs, insurance‐related barriers, and time‐related impacts, contributing to the subjective financial distress borne by patients.

**HOW MIGHT THIS CHANGE CLINICAL PHARMACOLOGY OR TRANSLATIONAL SCIENCE?**

Future research should focus on longitudinal evaluations of financial toxicity and aim to prioritize strategies to improve access to treatment while minimizing the financial burden. Healthcare providers should recognize that the financial burden relating to biological medicines and related subjective financial distress can directly influence treatment adherence, health outcomes, and patient well‐being.

The rising costs of medical treatments have brought increased attention to the concept of financial toxicity, which refers to the economic burden experienced by patients due to medication and other healthcare expenses and their accompanying consequences, such as behavioral effects resulting in nonadherence to treatment, diminished quality of life, in addition to material and psychosocial effects.^1^, ^2^, ^3^, ^4^, ^5^ First used in an academic context and initially documented in oncology,^6^ where patients often face high expenditures for life‐saving therapies, financial toxicity is now recognized as a broader issue affecting individuals with chronic illnesses who require long‐term medical interventions.^7^, ^8^, ^9^

Witte *et al*. (2019)^5^ conceptualized a framework for defining the aspects of financial toxicity, describing it as comprising both the objective financial burden of direct and indirect treatment costs and the subjective financial distress borne by patients. This subjective distress includes material (e.g. having to sell assets to afford treatment) as well as psychological and behavioral responses. Research by Zafar *et al*.^4^ and Ghazal *et al*.^10^ has further highlighted that the financial burden of medical treatments can lead to emotional and social stress, which, in turn, negatively impacts health outcomes.

Out‐of‐pocket expenses are a central factor in financial toxicity, with their impact varying across healthcare systems depending on insurance coverage, reimbursement systems, and patients' purchasing power.^8^, ^11^ Even in publicly funded systems with high reimbursement levels, patients may face substantial indirect treatment‐related costs, such as travel expenses related to accessing healthcare services and lost wages due to time away from work for treatment or medical appointments, while those with private insurance may encounter high deductibles and co‐payments.^4^ Certain populations, including individuals from lower income households, patients with several diseases, those relying solely on public insurance, in addition to young adults and women, are disproportionately affected by financial toxicity.^12^, ^13^, ^14^ Financial constraints have been shown to negatively influence medication adherence, with some patients skipping or lowering doses or avoiding prescriptions altogether.^3^ Moreover, the psychological distress related to financial toxicity can further impair both physical and mental well‐being.^7^, ^10^ Transparent communication between healthcare providers and patients regarding treatment costs has been linked to improved adherence and better health outcomes.^15^, ^16^

While high drug list prices of biological medicines contribute substantially to overall healthcare expenditure, their impact on the patient‐level financial burden varies across reimbursement systems. In countries that have placed annual expenditure ceilings on reimbursed drug spending, such as the Nordic countries, patients may reach the maximum annual out‐of‐pocket limit after a single purchase regardless of whether a medicine costs €800 or €8,000. In contrast, in insurance models with deductibles, co‐insurance, or specialty tier cost‐sharing (e.g., the U.S. or low‐to‐middle‐income (LMI) county settings), higher drug prices directly translate into higher out‐of‐pocket spending and increased financial toxicity.

Financial toxicity has become particularly relevant in the context of biological medicines, which are advanced, innovative therapies commonly used to treat cancer, autoimmune disorders, and chronic inflammatory diseases. Despite their therapeutic benefits, biological medicines are often significantly more expensive than traditional chemical drugs due to extensive research and development, complex manufacturing processes, and stringent regulatory requirements.^17^, ^18^, ^19^, ^20^, ^21^ Consequently, financial barriers may limit patient access and adherence to these treatments, exacerbating the burden of disease.^20^ Although financial toxicity can occur with any high‐cost therapy, biologics warrant specific attention because they are frequently placed on specialty tiers, have high list prices, their use is growing fast, and are frequently used for long periods of time across large patient groups. At the same time, we acknowledge that financial toxicity is driven by cost‐sharing structures rather than drug class alone. Our focus on biologics and biosimilars reflects their substantial and well‐documented contribution to patient out‐of‐pocket spending. While financial toxicity has been extensively explored in oncology, there is a lack of reviews that examine biologic medicines as a therapeutic class across disease areas. Our scoping review addresses this gap. A systematic review from 2022^22^ examined the prevalence of financial toxicity among patients with hematologic malignancies and found 20–50% experienced significant economic burdens, which were often heightened by the high costs of biologic therapies frequently used in these cancers. Younger, lower income, unemployed, or rural patients were found to be particularly vulnerable, as the high out‐of‐pocket expenses of biologics can compound losses from reduced work productivity, travel costs, and depletion of savings. The review also found substantial heterogeneity in how financial toxicity is measured and noted that reducing the financial strain related to treatment, including biologics, may even improve survival outcomes.

The introduction of biosimilars, which are highly similar to original biological medicines but typically less costly, offers a potential solution to reduce healthcare expenditures.^21^, ^23^, ^24^ However, their adoption in clinical practice remains inconsistent due to variations in reimbursement policies and patients' and healthcare providers' concerns regarding efficacy, safety, and the nocebo effect.^25^, ^26^, ^27^, ^28^ However, despite their lower list prices, biosimilars have not yet fully reduced the patient‐level financial burden, as many patients continue to face substantial out‐of‐pocket payments, percentage‐based co‐insurance, deductibles, and speciality‐tier cost‐sharing. These mechanisms often limit the extent to which biosimilar discounts translate into lower direct costs for patients.^20^ The uptake of biosimilars in clinical practice remains uneven across countries. In health systems with centralized purchasing and clear guidance on substitution, which are common in many social insurance models, biosimilars are adopted more quickly. In contrast, in systems with higher patient cost‐sharing and more fragmented reimbursement structures, uptake may be slower due to payer policies, formulary restrictions, and ongoing concerns among clinicians and patients regarding efficacy, safety, and the potential for nocebo effects.^29^ Moreover, patients' willingness to pay and ability to pay for biologic treatments, shaped by income levels and cost‐sharing requirements, further influence real‐world adoption patterns.

Given the growing reliance on biological medicines and their substantial contribution to rising pharmaceutical spending, many health systems, especially in Europe, North America, and several middle‐income countries, have identified a need to better understand their clinical and economic implications to inform reimbursement and policy decisions.

This scoping review aims to synthesize current evidence on financial toxicity connected to biological medicines, adopting the framework for financial toxicity from Witte *et al*.^5^ (**Figure** **1**). Additionally, this study aims to propose policy interventions to mitigate the impact of financial toxicity and to identify key research gaps.

**Figure 1:**
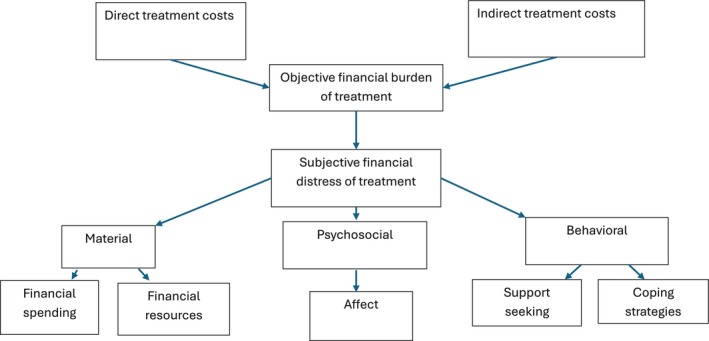
Framework of financial toxicity. The objective financial burden and subjective financial distress, adapted from Witte *et al*.

## METHODS

This scoping review was conducted following the methodological guidance of Arksey and O'Malley^30^ and the Joanna Briggs Institute^31^ and was reported in accordance with the PRISMA guideline.^32^ The study protocol was pre‐registered in the Open Science Framework (OSF).^33^

### Identification of relevant studies

A comprehensive literature search was conducted on October 10, 2024 using bibliographic databases: Scopus, Medline via Ovid, Web of Science, CINAHL, and EMB Reviews. Search strings were developed based on the Population, Concept, and Context (PCC) framework.^31^ The detailed search strategies were further guided by the framework by Witte *et al*.,^5^ and were built around the keywords *financial toxicity, biologics*, *biologicals, treatment*, and their synonyms. We focused our search on the term ‘financial toxicity’ and its near‐synonyms to optimize specificity for studies explicitly conceptualizing medicine‐related financial hardship within Witte's framework^5^ in the context of biologic medicines. We acknowledge that related constructs (e.g., catastrophic health expenditure, cost‐related nonadherence, out‐of‐pocket burden) capture overlapping dimensions of affordability; however, including all such terms would have risked substantial conceptual heterogeneity. A detailed search strategy from Scopus is presented in the Supplemental Material S1.

### Study selection

**Figure 2:**
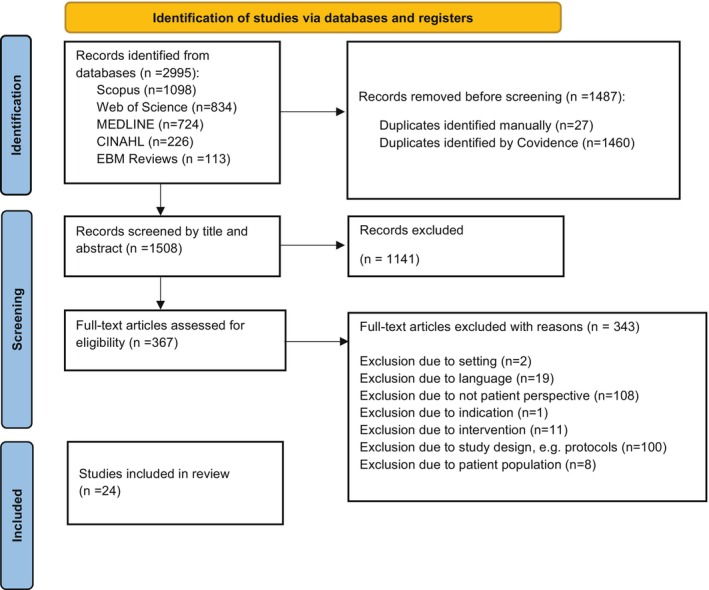
PRISMA flow diagram. Illustration of the screening, inclusion and exclusion process.

All articles were screened for relevance based on the predefined eligibility criteria. Studies were included if they focused on patients of any age using originator biological medicines (reference products) or biosimilars; investigated or described the use of biological medicines and/or biosimilars; reported outcomes of financial toxicity, including cost of treatment from the patient's perspective, out‐of‐pocket expenses, and/or the subjective financial distress related to material, psychosocial and behavioral effects; were observational in design (retrospective, prospective, or cross‐sectional), or were randomized clinical trials. Articles including expert opinions, pharmacoeconomic evaluations, and narrative reviews that did not directly meet the above eligibility criteria were excluded. In addition, studies were excluded if the population (patients), indication (requiring use of biological medicines), setting (patient‐level focus), intervention (biological medicines), or outcome (costs to the patient) did not meet the inclusion criteria. Systematic reviews were not excluded; however, none were identified that met the inclusion criteria. The search was restricted to peer‐reviewed journal articles published in English over the past 20 years; the publication years of the identified studies ranged from 2008 to 2023 to ensure locating all relevant references around the term, which was first used, albeit outside academic context, in 2009.^34^ The Covidence software was used for article screening, performed by two independent reviewers (KY and AS). The potential conflicts were solved by a third reviewer (LS). The search yielded 2,955 articles, and ultimately, 24 articles fulfilled all eligibility criteria and were included in the final analysis. **Figure** **2** illustrates the selection process in a PRISMA flow diagram.

### Data extraction and synthesis

Data extraction, conducted using a customized template within the Covidence platform, was based on the objectives of the present scoping review. Extracted information included publication details, study design, sample characteristics, definitions and measurements of financial toxicity, and key findings corresponding to the study objectives. The reported expenditures were converted to U.S. dollars ($) using the European Commission's October 2025 exchange rates.^35^ Data were extracted by one reviewer (KY) and subsequently assessed for accuracy by a second reviewer (AS).^31^ The extracted data were then synthesized descriptively, with findings organized by country, study design, patient population, intervention type, and reported outcomes. Where possible, we standardized reported direct costs to Per Patient Per Year (PPPY) values based on information available in the original studies. A quality assessment of the included studies was not conducted.

## RESULTS

### Study characteristics

High heterogeneity was observed across studies in terms of geographic origin, methodological design, patient populations, interventions, and outcome measures. When reporting results, we use “financial burden” when referring to studies reporting only objective cost measures and reserve the term “financial toxicity” for findings addressing the broader conceptual framework. The studies originated from a diverse set of countries, encompassing both high‐income and low‐to‐middle‐income country (LMIC) settings, including, for example, the United States, Malaysia, China, Germany, and Saudi Arabia. The majority of studies employed observational designs, with retrospective analyses being the most prevalent (*n* = 10), often utilizing large administrative or insurance claims databases to evaluate cost outcomes, adherence, and coverage patterns for biological medicines.^36^, ^37^, ^38^, ^39^, ^40^, ^41^, ^42^, ^43^, ^44^, ^45^ Cross‐sectional survey studies (*n* = 6) provided patient‐reported data on the financial burden, with sample sizes ranging from 200 to 2,737 participants.^46^, ^47^, ^48^, ^49^, ^50^, ^51^ These studies offered insights into the challenges with the out‐of‐pocket costs spending and affordability of medicines. Three studies^52^, ^53^, ^54^ adopted a prospective observational design, while five employed modeling approaches to simulate out‐of‐pocket expenses under various healthcare scenarios, such as Medicare Part D coverage or transportation‐related costs.^55^, ^56^, ^57^, ^58^, ^59^

The most frequently studied patient populations were individuals with rheumatoid arthritis and psoriasis, comprising approximately one‐third of the included studies, as detailed in **Table** **1**. A subset of studies (*n* = 4) focused on inflammatory bowel diseases, including Crohn's disease and ulcerative colitis.^36^, ^39^, ^54^, ^57^ Other conditions included systemic lupus erythematosus (*n* = 2),^38^, ^55^ atopic dermatitis (*n* = 1),^50^ severe asthma (*n* = 1),^41^ and juvenile idiopathic arthritis (*n* = 1),^48^ with the latter representing pediatric populations. Two studies examined the financial implications of immune checkpoint inhibitors and CAR T‐cell therapies.^53^, ^58^ Collectively, the studies represented data from over 3.1 million patients.

**Table 1:** Objective financial burden (adapted from Witte *et al*.^5^) connected with biological medicine use.

Study reference	Country and time of study	Methods and data	Population	Biological medicines	Direct patient costs reported [PPPY]	Indirect patient costs reported
AlRuthia *et al*. (2019)^36^	Saudi Arabia (March 2016 – October 2018)	Single‐center retrospective chart review using electronic health records	Patients with CD (*n* = 258) and UC (*n* = 249)	Ustekinumab, vedolizumab	$7,030–23,247 [ustekinumab $7,030 – vedolizumab $23,247]	N/A
Balogh *et al*. (2014)^46^	Hungary (September 2012–May 2013)	Cross‐sectional survey and cost calculation using societal perspective	Adults with moderate to severe psoriasis attending dermatology clinics (*n* = 200)	Adalimumab, etanercept, infliximab, ustekinumab	Avg. annual biological medicine per patient cost $15,965 [$15,965]	$2,730 (HCA); $668 (FCA)
Blauvelt *et al*. (2022)^37^	United States (March 2016 – October 2019)	Retrospective cohort analysis using IBM MarketScan® claims databases	Adults with psoriasis receiving IXE (*n* = 471) or SEC (*n* = 990)	Ixekizumab, secukinumab	$336/month (IXE), $301/month (SEC) [IXE $4,032, SEC $3,612]	N/A
Clarke *et al*. (2020)^38^	United States (January 2010–December 2014)	Retrospective analysis of IBM MarketScan® databases (claims data)	Adults (≥18 years) with SLE treated with antimalarials, selected biologics, immuno‐suppressants, or systemic glucocorticoids (*n* = 9,033)	Abatacept, Belimumab, Rituximab	N/A	N/A
Elsisi *et al*. (2024)^55^	Malaysia (transition probabilities based on data from 2006 to 2010)	Cost‐of‐illness model with state transition approach validated by Delphi panel	Adults (≥15 years) with systemic lupus erythematosus (SLE) (*n* = 18,121)	Rituximab	N/A	N/A
Hamuryudan *et al*. (2016)^47^	Turkey (May 2011–August 2012)	Cross‐sectional patient interviews with cost data collected via standard questionnaire	Adults with RA receiving care in rheumatology outpatient clinics (*n* = 689)	Medicines not specified but biologicals included	Included in $4,169 (mean annual, all medicine types) [$4,169]	Work loss: $728; caregiver: $960; equipment/home adaptation: $2,517
Karaca‐Mandic *et al*. (2010)^56^	United States (2000–2005)	Multivariate regression analysis using claims data from 35 employers, 176 health plans	Patients with RA covered under employer health plans (*n* = 8,557)	Etanercept, adalimumab, infliximab	Avg. Annual cost $632 up to $1,780 (self‐administered medicines filed under pharmacy claims); Avg. $2,253 up to $7,362 (medical claims) [$632 – $1,780; $2,253 – $7,362]	N/A
Kuhlmann *et al*. (2016)^48^	Europe: Germany, Italy, Spain, France, UK, Bulgaria, Sweden(2012)	Multinational cross‐sectional study using self‐reported questionnaires	Children/adolescents with JIA and their caregivers (*n* = 317)	Medicines not specified but biologicals included	Included in $759/year (OOPs including travel, OTC, devices) [$759]	Caregiver productivity loss, home care costs discussed
Min *et al*. (2023)^49^	China (July 2021 – December 2022)	Cross‐sectional survey using questionnaire‐based data collection	Psoriasis patients attending a dermatology clinic, aged 18–80, treated with biologicals (*n* = 286) or non biologicals (*n* = 195)	Secukinumab, adalimumab, istekinumab, ixekizumab	$2,192/year (94.3% of total medical costs) [$2,192]	$132/year (caregiver + patient loss)
Mohr *et al*. (2022)^50^	Germany (August 2017 – June 2019)	Cross‐sectional survey‐based analysis with routine clinical data	Adults with atopic dermatitis receiving routine care at 40 dermatology clinics (*n* = 815)	Medicines not specified but biologicals included	Included in $383/year per patient (total OOP) [$383]	$629/year per patient (missed work days)
Park *et al*. (2020)^39^	United States (2007–2016)	Retrospective cohort using Optum claims data (2007–2016)	Commercially insured and Medicare Advantage IBD patients (*n* = 52,782)	Medicines not specified but biologicals included	$2,758/year (includes deductibles, co‐pays, etc.) [$2,758]	Estimated >$2,490/year via productivity loss
Polinski *et al*. (2009)^40^	United States (January – July 2006)	Analysis of Medicare Part Dplan formularies and benefit designs related to RA biological therapies	Medicare beneficiaries with RA	Etanercept, adalimumab	Varied by plan; often substantial due to specialty tier placement; biological medicines often placed in tiers requiring higher cost‐sharing	N/A
Rankala *et al*. (2021)^57^	Finland (September 2015 – August 2016)	Survey and registry‐based analysis using HCA	Employed patients with CD or UC (*n* = 320)	Infliximab, adalimumab, vedolizumab, golimumab	N/A	Loss of productivity due to absenteeism: $878/patient/year; and presenteeism (working when ill): $883/patient/year
Rao *et al*. (2017)^54^	United States (January 2009 – December 2013)	Observational analysis of prospectively collected data from a registry	Adult patients with confirmed CD from University of Pittsburgh Medical Center registry (*n* = 243)	Medicines not specified but biologicals included	N/A	N/A
Reibman *et al*. (2021)^41^	United States (January 2012 – June 2019)	Retrospective claims data analysis (IBM MarketScan) from 2013 to 2018	Patients with severe asthma treated with omalizumab, mepolizumab, reslizumab, or benralizumab (*n* = 3,262)	Benralizumab, mepolizumab, omalizumab, reslizumab	Included (co‐payments, deductibles, co‐insurance) but not broken down numerically	N/A
Schaefer *et al*. (2015)^42^	United States (January – May 2012)	6‐month retrospective chart review and patient survey at 8 community‐based dermatologists and 1 primary care physician sites	Adults with moderate to severe plaque psoriasis actively seeking care (*n* = 200)	Alefacept, infliximab, Ustekinumab, etanercept, adalimumab	$15,349 over 6 months (direct costs) [$30,698]	$2,856 over 6 months
Shi *et al*. (2018)^43^	Taiwan (January 2009 – December 2013)	Retrospective cohort using national health insurance claims data	Biologic‐naïve RA patients initiating bDMARDs (*n* = 818)	Adalimumab, etanercept	Total medication costs $17,252, $13,010, $11,560 from year one to three [Y1: $17,252, Y2: $13,010, Y3: $11,560]	N/A
Snyder *et al*. (2021)^58^	United States (2011–2015)	Geographic Information System methods combined with economic modeling	Patients with relapsed/refractory DLBCL eligible for CAR T‐cell therapy (*n* = 3,922)	Axicabtagene ciloleucel, tisagenlecleucel	N/A	Estimated travel costs (mean, weighted) in different scenarios patient: $2,525 – 3,696, care giver: $1,902 – 2,660
Takahashi *et al*. (2017)^44^	Japan (April 2015–March 2016)	Retrospective analysis of patient records from one outpatient clinic	Psoriasis vulgaris patients receiving topical or systemic treatment (*n* = 148)	Secukinumab, Ustekinumab, Adalimumab	$4,478 – 6,305/year for biological medicines [$4,478 – $6,305]	N/A
Yazdany *et al*. (2015)^51^	United States (January 2013)	Cross‐sectional analysis of Medicare Part D formularies	Medicare beneficiaries with RA (*n* = 2,737)	Abatacept, adalimumab, anakinra, certolizumab, etanercept, golimumab, infliximab, rituximab, tocilizumab	$3,692 – 3,777/year before reaching catastrophic coverage phase, after which patients pay 5% of drug costs [$3,692 – $3,777]	N/A
Yazdany *et al*. (2018)^59^	United States (June 2017)	Simulated cost modeling using Medicare Plan Finder data and ASP	Patients with RA eligible for Medicare Part D	Infliximab (originator and biosimilar)	$6,730/year (infliximab) under Medicare Part D [$6,730]	N/A

ASP, Average Sales Price; bDMARDs, Biologic disease‐modifying antirheumatic drugs; CAR T, Chimeric antigen receptor T‐cell therapy; CD, Crohn's disease; DLQI, Dermatology Life Quality Index; DLBCL, Diffuse large B‐cell lymphoma; FCA, Friction Cost Approach; HCA, Human Capital Approach; IBD, Inflammatory bowel disease; ICIs, Immune checkpoint inhibitors; IXE, Ixekizumab; JIA, Juvenile idiopathic arthritis; OOP, Out‐of‐pocket; OTC, Over‐the‐counter; RA, Rheumatoid arthritis; SEC, Secukinumab; SLE, Systemic lupus erythematosus; UC, Ulcerative colitis.

The most commonly evaluated biological agents were adalimumab, infliximab, ustekinumab, and secukinumab. Eight studies specifically investigated biosimilars,^39^, ^44^, ^45^, ^49^, ^50^, ^51^, ^55^, ^56^ though most employed modeling rather than real‐world comparative designs. None of the studies assessed patient experiences related to switching from originator biological medicines to biosimilars. Nonbiological comparators, such as conventional disease‐modifying antirheumatic drugs (DMARDs) and topical agents, were occasionally included as baseline treatments.

Despite the methodological and contextual variability across studies, several consistent patterns emerged. Patients frequently reported an objective financial burden from biological therapies, including high out‐of‐pocket expenditures and limited insurance coverage.

These financial stressors attributed to a subjective financial distress manifesting as psychological consequences, as well as treatment adherence challenges. These patterns are detailed below.

### Objective financial burden: Direct treatment costs (out‐of‐pocket)

Out‐of‐pocket (OOP) costs were the most consistently reported metric of financial toxicity, with 22 of the 24 studies addressing this aspect related to direct treatment costs (**Table** **1**). These costs, primarily co‐payments and deductibles, were identified as key contributors to direct treatment costs attributing to financial toxicity in patients using biological medicines. Co‐payments were reported in 18 studies,^36^, ^37^, ^38^, ^39^, ^40^, ^41^, ^42^, ^44^, ^45^, ^47^, ^48^, ^49^, ^51^, ^52^, ^53^, ^56^ while deductibles were discussed in 12 studies.^36^, ^37^, ^38^, ^39^, ^40^, ^41^, ^45^, ^49^, ^51^, ^53^, ^56^, ^59^ These expenses were often linked to specialty tier placements in insurance formularies, regional co‐insurance policies, and Medicare Part D structures in the U.S.

Studies consistently showed that high co‐payments and deductibles were barriers to treatment initiation or adherence,^39^, ^51^, ^56^ and in some cases, led to delays, switching, or complete discontinuation of biological therapies.^49^, ^53^ Even biosimilars, despite their lower list prices, remained financially inaccessible due to high co‐insurance requirements. Insurance‐related barriers were reported in 16 studies.^36^, ^37^, ^38^, ^39^, ^40^, ^41^, ^42^, ^44^, ^45^, ^49^, ^50^, ^51^, ^53^, ^55^, ^56^, ^59^ These included high co‐insurance rates and specialty tier structures in the United States,^38^, ^39^, ^42^, ^45^, ^51^, ^59^ and limited coverage for biosimilars in such countries as India and China.^49^, ^53^ Such barriers were frequently linked to delays in biological medicine treatment initiation or therapy discontinuation.

### Objective financial burden: Indirect treatment costs

Ten studies^39^, ^42^, ^45^, ^47^, ^48^, ^49^, ^50^, ^52^, ^55^, ^57^ reported indirect costs, which included lost income and reduced productivity. Additionally, studies reported travel expenses and increased caregiver time. In many inflammatory conditions, indirect costs tend to reflect disease‐related impairment more than administration costs. Because several included studies did not differentiate these contributors, attribution should be interpreted cautiously. In studies based in LMICs,^52^, ^55^ indirect costs could exceed direct treatment costs. Assessment methods of indirect costs varied, including human capital approaches^46^, ^57^ and patient surveys.^47^, ^48^, ^50^

### Subjective financial distress

Six studies^45^, ^48^, ^49^, ^50^, ^54^, ^57^ evaluated the psychological effect relating to the objective financial burden connected to high treatment costs. The subjective financial distress reported in the included studies is presented in **Table** **2**. Instruments used to measure this included the Dermatology Life Quality Index (DLQI), EuroQol 5‐Dimension (EQ‐5D), Visual Analog Scales (VAS), and the Work Productivity and Activity Impairment Questionnaire (WPAI). These studies reported that subjective financial distress was related to reduced quality of life and increased anxiety, which, in turn, impaired daily functioning.

**Table 2:** Subjective financial distress (adapted from Witte *et al*.^5^) connected with biological medicines use

Study reference	Material: Financial spending	Material: Use of financial resources	Psychosocial effects	Behavioral changes: support seeking	Behavioral changes: coping care and coping lifestyle
Blauvelt *et al*. (2022)^37^	N/A	Total all‐cause costs (mean ± SD): IXE users = $6,183 ± 3,614 and SEC users = $5,612 ± 2,926	N/A	N/A	Measured: 39.1% of IXE vs. 31.6% of SEC users were highly adherent (PDC ≥80%)
Elsisi *et al*. (2024)^55^	N/A	N/A	Caregiver and patient stress/anxiety mentioned but not measured	N/A	N/A
Karaca‐Mandic *et al*. (2010)^56^	NA	N/A	N/A	N/A	Initiation of biologicals lower in high OOP burden households
Kuhlmann *et al*. (2016)^48^	N/A	N/A	Zarit caregiver burden scale used; high perceived burden in some countries	N/A	EQ‐5D and VAS scores collected; mean EQ‐5D index 0.83
Min *et al*. (2023)^49^	N/A	N/A	N/A	N/A	Cost as barrier to biological medicine use;DLQI scores reported: BT group had better HRQoL than non‐BT group
Mohr *et al*. (2022)^50^	N/A	N/A	N/A	N/A	DLQI, EQ‐VAS, PBI measured; correlated with cost burden
Philips *et al*. (2023)^53^	N/A	N/A	N/A	Family support and aid used when available	Not measured numerically, but inferred from discontinuation causes
Polinski *et al*. (2009)^40^	N/A	N/A	N/A	N/A	Potential for reduced adherence due to high out‐of‐pocket costs
Rankala *et al*. (2021)^57^	N/A	N/A	N/A	N/A	Negative correlation between IBDQ scores and presenteeism/absenteeis m costs (assessed with IBDQ32)
Rao *et al*. (2017)^54^	N/A	N/A	Psychiatric co‐morbidities present in 32% of patients; more common in the worsening disease group	N/A	N/A
Reibman *et al*. (2021)^41^	N/A	N/A	N/A	N/A	Discontinuation rate ~ 36%; 52.1% adherence (≥80% administration ratio); persistence lower in uncontrolled patients
Shi *et al*. (2018)^43^	N/A	N/A	N/A	N/A	36% had low adherence (<80%); 66% were classified as inadequate responders
Snyder *et al*. (2021)^58^	Modeled indirectly via percentage of income	N/A	N/A	Mention of manufacturer assistance programs, but not evaluated	N/A
Yazdany *et al*. (2015)^51^	N/A	Not directly reported, but financial trade‐offs implied. Medicare does not allow copay coupons, limiting patient options. Discussed vianational survey: 12% reduced spending on basic needs	N/A	N/A	National data: 1 in 6 patients with RA reduced treatment due to costs
Zhang *et al*. (2023)^45^	N/A	Patients may go without essentials to afford treatment	Described in context of financial burden from increased ASP	N/A	Cost‐related nonadherence and increased loss to follow‐ up identified. Use of off‐label bevacizumab; step therapy discussed as a payer strategy

Abbreviations: ASP, Average Sales Price; BT, Biological therapy; DLQI, Dermatology Life Quality Index; EQ‐5D, EuroQol 5‐Dimension scale; EQVAS, EuroQol Visual Analog Scale; HRQoL, Health‐Related Quality of Life; IBDQ, Inflammatory Bowel Disease Questionnaire; IXE, Ixekizumab; OOP, Out‐of‐pocket; PBI, Patient Benefit Index; PDC, Proportion of Days Covered; RA, Rheumatoid arthritis; SEC, Secukinumab; VAS, Visual Analog Scale.

Eleven studies^37^, ^38^, ^39^, ^40^, ^41^, ^43^, ^45^, ^51^, ^53^, ^56^, ^58^ documented care‐related behavioral changes, which included treatment nonadherence or discontinuation due to subjective financial distress. High‐tier cost‐sharing, particularly under Medicare Part D in the U.S.,^45^, ^51^, ^56^ discouraged treatment initiation and led to skipped doses. Coping strategies such as seeking financial aid or relying on family support were reported in two studies.^53^, ^58^ Additionally, material consequences discussed included sacrificing everyday basic needs (e.g., food, housing, or transportation), which resulted in delays in receiving care.^45^, ^51^

## DISCUSSION

This scoping review synthesis reveals the complex and multifaceted nature of financial toxicity connected to the use of biological medicines, encompassing direct and indirect treatment costs, insurance‐related barriers and time‐related impacts, contributing to the subjective financial distress borne by patients. While biological medicines have transformed the treatment of chronic and immune‐mediated diseases, their financial implications can significantly compromise patient access, adherence, therapeutic outcomes, and overall quality of life.

Patient‐level financial toxicity emerges from the combined influence of medicine prices, the design of reimbursement mechanisms, and the range of nonmedical expenses incurred during treatment. Financial toxicity varies markedly by country context. In health systems characterized by high cost‐sharing, such as the U.S., co‐payments and deductibles play a fundamental role in determining both the timing and magnitude of patients' out‐of‐pocket payments and deductibles. Making reimbursement designs, not medicine prices alone, a key driver of financial toxicity. Conversely, in social insurance–based systems, such as in many Nordic countries, payment ceilings, exemptions, and other protective mechanisms may reduce, but do not fully eliminate, patients' medicine‐related financial burdens.

Direct out‐of‐pocket (OOP) expenditures, primarily co‐payments and deductibles, were consistently identified as the most immediate and measurable source of financial burden. The reviewed studies demonstrated that high OOP costs were related, for example, to reduced treatment initiation, skipped doses, and therapy discontinuation. Even biosimilars, which are intended to reduce costs, often remained financially inaccessible despite reimbursement policies. These costs were particularly impactful in healthcare systems with tiered insurance structures, such as Medicare Part D in the United States, where specialty drugs often incur higher co‐insurance rates. These cost‐sharing mechanisms are designed to mitigate moral hazard, the tendency for insured individuals to overutilize healthcare services when shielded from the full cost, and thus, controlling payer expenditures.^60^, ^61^, ^62^ Simultaneously, national reimbursement systems aim to ensure equitable access to essential medications.^63^ However, even minor policy changes, such as increased deductibles or stricter eligibility criteria, can directly affect patients' ability to initiate and maintain biologic treatment.^61^, ^64^ For individuals requiring long‐term, high‐cost therapies, these financial barriers often result in delayed initiation, reduced adherence, or complete discontinuation.^56^ However, these patterns reflect reimbursement pathways rather than drug class alone, and in some health systems hospital‐provided biologics may involve lower patient cost‐sharing than chemically synthesized medicines dispensed in retail pharmacies. Insurance‐related barriers were prevalent across the studies, including limited coverage for biosimilars, rigid reimbursement criteria, and high co‐insurance rates. These systemic issues disproportionately affect vulnerable populations, including those with lower socioeconomic status or limited insurance literacy. The complexity of navigating insurance approvals and formulary restrictions often leads to delays in treatment initiation or complete abandonment of therapy^65^ Such barriers not only exacerbate financial toxicity but also contribute to health disparities.

Indirect costs form an essential component of financial toxicity and substantially shape patients' overall burden. Indirect costs represent a critical component intrinsic to the disease process in chronic conditions, such as rheumatoid arthritis, psoriasis, and inflammatory bowel disease, causing fatigue, pain, and functional limitations that impair a patient's ability to work, travel, or engage in daily activities. These include lost income due to work absenteeism, reduced productivity, transportation expenses, and caregiver time. Importantly, these burdens are not solely attributable to the treatment itself but are often caused by the disease. Moreover, biological medicines have been shown to decrease indirect costs in patients, attributing to fewer days on sick leave and longer working careers overall due to superior clinical outcomes.^66^, ^67^

Treatment‐related indirect costs also encompass the concept of time toxicity, which refers to the time patients must spend managing their treatment and healthcare needs. This includes the time spent traveling to and receiving infusions, waiting for specialist appointments, dealing with insurance approvals, managing adverse effects, and attending routine monitoring. It also extends to the time caregivers dedicate to supporting patients during flare‐ups or appointments. These time demands can disrupt work, reduce productivity, and add to the overall indirect costs of treatment, especially for those with limited access to care or support systems. Time demands are especially burdensome for patients with limited mobility, those living in remote areas, or individuals with inflexible work schedules.^68^ In LMICs, where healthcare infrastructure may be less accessible and social support systems are limited, both disease‐driven and time‐related indirect costs can exceed the direct costs of medication.

In this review, the patients' financial experiences varied significantly depending on disease type, clinical context, and treatment options. Biological therapies are commonly prescribed for chronic inflammatory, immune‐mediated, and oncological conditions, all of which typically require sustained or lifelong treatment. Studies in dermatology and rheumatology often included patient‐reported outcomes such as the Dermatology Life Quality Index and work productivity measures,^42^, ^50^ while oncology‐focused studies relied more heavily on modeled projections and system‐level cost analyses.^58^ Studies on diseases with established biosimilar markets, such as rheumatoid arthritis and psoriasis, frequently discussed switching strategies and price comparisons,^44^ whereas studies on conditions with limited biosimilar availability emphasized access and affordability barriers.^55^, ^58^ Together, these findings illustrate that disease‐specific financial experiences are shaped not only by treatment characteristics but also by broader system‐level factors, including insurance design and reimbursement policies.

This scoping review followed established methodological guidelines.^30^, ^31^, ^32^ Nonetheless, several limitations should be acknowledged. First, restricting the review to English‐language publications may have excluded relevant studies in other languages. Second, focusing exclusively on the patient perspective excluded system‐level analyses that may offer valuable insights into broader economic impacts. Third, studies that addressed reimbursement systems or policy effects without explicitly referencing financial toxicity may have been missed. Including terms such as “catastrophic health costs” would have broadened the search to capture general health expenditure studies, particularly from LMIC contexts, but these works typically apply macro‐level financial‐risk protection frameworks rather than patient‐level financial toxicity as conceptualized by Witte *et al*.^5^ These terms were excluded from the search strategy to maintain conceptual specificity and focus on treatment‐related financial toxicity associated with biological therapies. Despite these limitations, a comprehensive search strategy across five major databases and clearly defined inclusion criteria helped mitigate potential bias.

While the economic burden of biological therapies is well‐documented in terms of direct and indirect costs, the psychosocial and behavioral aspect of financial toxicity remains largely underexplored. Only a minority of studies assessed the psychosocial impacts of treatment‐related financial distress, and even fewer examined both economic and psychological outcomes concurrently. The ECHO model (Economic, Clinical, and Humanistic Outcomes)^69^ could provide a useful framework to highlight this imbalance. Although biological therapies are rigorously evaluated for clinical efficacy and population‐level cost‐effectiveness,^70^ patient‐level economic burden and humanistic outcomes, such as emotional well‐being, functional status, and quality of life, are often overlooked. This gap is further compounded by limitations in current assessment tools. While several validated instruments exist to measure the overall burden of medicine use, such as HRQoL instruments, they typically focus on practical, psychological, or informational aspects. Generic HRQoL instruments (e.g., EQ‐5D) may not fully capture the financial burden borne by patients. Future work should validate fit‐for‐purpose financial toxicity measures and examine their responsiveness to policy changes affecting patient cost‐sharing.

The cumulative evidence suggests that addressing financial toxicity requires a comprehensive approach. Studies by Yazdany *et al*.^51^ and Zhang *et al*.^45^ documented that some patients even sacrifice basic needs, such as food, housing, and transportation, to afford treatment, reflecting the profound ways in which financial burden shapes everyday life choices. In response, many adopted coping mechanisms, such as switching medications, seeking financial aid, or relying on family support; however, these measures were commonly insufficient to fully mitigate the burden. These findings underscore the need to view financial toxicity not only as an economic challenge but as a significant psychosocial determinant of health.

While the diversity of healthcare systems and economic contexts limits the generalizability of the results of financial toxicity studies, it also enriches the findings of this present study by reflecting a wide range of patient experiences. Ultimately, this review contributes to a more nuanced understanding of the financial, psychosocial, and systemic challenges linked to biological medicine use from the patient's perspective. Real‐world evaluations of biosimilar switching, patient‐centered cost‐mitigation strategies, and integration of mental health support are essential to improving the overall care experience.

Directed by Witte's framework,^5^ our findings suggest that policy efforts should aim at reducing both the objective financial burden and the subjective financial distress connected to biological therapies. Evaluating and strengthening reimbursement systems through lowering co‐payments, income‐based caps, reduced deductibles, installment‐based payment options (e.g.^71^), or simplified access criteria can directly ease the direct out‐of pocket burden borne by patients and contribute to more rational treatment practices. As an additional example, improved service organizations and medication schemes with wider availability of subcutaneous formulations or decentralized administration may reduce time and productivity losses that contribute to indirect costs.^72^, ^73^ Patient‐centered support systems, that is, practical mechanisms such as financial navigation, cost transparency resources and tools, patient education initiatives, and assistance programs may help alleviate material, psychosocial, and behavioral components of subjective financial distress and contribute to more equitable access to biological medicines. Pharmacists and other healthcare professionals have an important role in providing patients with this information, in addition to medicine‐focused medication counseling.

Future research is needed to address several gaps identified in this review, including the lack of longitudinal studies tracking changes in both objective financial burden and subjective financial distress over time, limited evidence evaluating the effects of reimbursement reforms or biosimilar uptake policies on patient costs, and the scarcity of studies examining financial toxicity across varied health system contexts. Further research should focus on examining interventions such as reducing co‐payments or deductibles, improving access to biosimilars, increasing financial navigation support, or enhancing cost transparency to determine which policy and organizational approaches most effectively reduce financial toxicity. Reducing patients' out‐of‐pocket spending does not make expenses disappear; it reallocates them across caregivers, providers, insurers, payers, and eventually taxpayers. Because these shifts touch every corner of the health system, such policies must be designed with precision to ensure that improved access to medicines does not come at the expense of equity or sustainability.

## FUNDING

This study was funded by the Strategic Research Council (SRC) established within the Academy of Finland (project 345388). Open access funding was provided by the University of Helsinki Library.

## CONFLICT OF INTEREST

The authors declared no competing interests for this work.

## AUTHOR CONTRIBUTIONS

L.S.M.S. and K.Y. wrote the manuscript; L.S.M.S., K.L. and A‐R.H. designed the research; K.Y., L.S.M.S., K.L. and A‐R.H. performed the research; K.Y., A.S. and L.S.M.S. analyzed the data.

## Supporting information


Data S1.
