# Supplementary material for: Financial Toxicity Associated with Biological Medicines: A Scoping Review
Source: Clin Pharmacol Ther. 2026 Apr 3;120(1):81–93. doi: 10.1002/cpt.70278 (PMC13264454; doi:10.1002/cpt.70278)
Supplement: Supplementary file 1 — Data S1. [file CPT-120-81-s001.docx]

Search string for Scopus:

( economic* OR financ* OR cost* OR expen* ) W/3 ( toxicity* OR burden* OR pollution* OR stress* OR distress* OR challenge* OR problem* )

( biosimilar* OR {biologic} OR {biologics} OR ( biologic* W/1 ( medic* OR drug* OR treatment* OR therap* ) )
